# Apparent Association of Insulin With Interleukin-6 (IL-6) in Severe COVID-19 Patients Having Chronic Disease Comorbidities

**DOI:** 10.7759/cureus.23790

**Published:** 2022-04-03

**Authors:** Fatimah A Nouh, Hajir Othman, Enass K Gwarsha, Agila A Elbadry, Akram Alabdali, Idris F Barassi, Salem Elamary, Abdelsalam A Elbadry, Farag A Elshaari

**Affiliations:** 1 Department of Biochemistry, Faculty of Medicine, University of Benghazi, Benghazi, LBY; 2 Department of Basic Medical Science, Faculty of Dentistry, University of Benghazi, Benghazi, LBY; 3 Department of Physiology, Faculty of Medicine, University of Benghazi, Benghazi, LBY; 4 Department of Internal Medicine, Hawari General Hospital, Benghazi, LBY; 5 Department of Laboratory Management, Faculty of Biomedical Sciences, University of Benghazi, Benghazi, LBY; 6 Department of Histology, Faculty of Medicine, University of Benghazi, Benghazi, LBY; 7 Department of Laboratory Medicine, Hawari General Hospital, Benghazi, LBY; 8 Department of Internal Medicine, Faculty of Medicine, University of Benghazi, Benghazi, LBY

**Keywords:** hypertension, diabetes, covid-19 comorbidity, interleukin 6, inflammatory marker, corona virus

## Abstract

Background

The severe acute respiratory syndrome caused by the novel coronavirus (COVID-19), due to its fast spread, is a disease with global health, social and economic burden. This is complicated by its high morbidity and mortality among those with medical comorbidities and older adults. During the outbreak in Libya, intensive care facilities were overwhelmed by the number of patients requiring special care. Admission to such facilities was reserved for severe cases showing low blood oxygen levels. Due to the inflammatory process in COVID-19, we believed it was essential to evaluate the outcome of inflammation reflected in the changes in interleukin-6 (IL-6) and insulin.

Objective

To study the changes in IL-6 and insulin during the course of the disease, if an association between them exists, and whether this association changes following seven days of treatment.

Method

We analyzed the data of 60 patients diagnosed with COVID-19 and admitted to the hospitals' Intensive Care Units (ICU) in the eastern part of Libya. The study was initiated on January 18th and concluded on March 22, 2021. Samples for the analysis were collected on the first day of admission and after seven days of hospitalization for patients who survived till the selected day. The collected samples were used to analyze IL-6 as an indicator of change in inflammation and insulin as a potential anti-inflammatory modulator. In addition, the association of insulin with IL-6 was statistically tested.

Results

Diabetes and hypertension, the most commonly observed chronic diseases in Libya, were found to represent the highest comorbidities among the ICU patients included in this study. Nonetheless, other diseases affected a smaller proportion of them, ranging from two patients for malignancy to 10 patients for cardiovascular disease. In addition, both age and gender showed differences in the number of ICU hospitalized patients and the death tally among them.

The study showed that the IL-6 level was on the rise during the course of COVID-19, whereas that of insulin was on the decrease. The two variables showed an association for admission day samples as well as for samples after seven days of ICU hospitalization.

Conclusion

Although, IL-6 appears to play a predictive role in the development and outcome of severe COVID-19, along with other biochemical and clinical findings it could serve as an indicator of the disease outcome. On the other hand, the role of insulin as a complementary factor for alleviating inflammation remains to be fully understood and requires further research. There is a pressing necessity for establishing the mechanism through which insulin is associated with inflammation modulatory pathways, in particular through the pathways involving IL-6.

## Introduction

Acute respiratory distress syndrome (ARDS) due to severe acute respiratory syndrome coronavirus 2 (SARS-CoV-2) was first diagnosed in Wuhan, China (December 2019), as a group of cases presented with atypical pneumonia, multi-organ failure, septic shock, disseminated intravascular coagulation, and in many cases, the ultimate death [1]. The WHO declared the outbreak a pandemic on March 11, 2020.

At the time of preparation of this manuscript, more than 312,173,462 cases and more than 5,501,000 deaths had been reported worldwide. In Libya so far 394,470 cases and 5,805 deaths were reported by the local authorities and the numbers are on the rise on a day-to-day basis [2].

The high fatality rate, especially in older patients in whom comorbidities including diabetes, cardiovascular disease, hypertension, obesity, and chronic obstructive pulmonary disease are common, makes the burden on healthcare givers and on patients heavier [3, 4].

The COVID-19 ARDS is associated with systemic hyper inflammation, believed to be the outcome of macrophage activation syndrome. Patients reaching this stage of the disease have features of the well-defined cytokine storm. Systemic levels of inflammatory biomarkers such as IL‐6, C-reactive protein (CRP), serum ferritin, coagulation index, D-dimer, TNF-α, IL-1β, and CXC-chemokine ligand 10 are well-established cytokine storm indicators. These are believed to lead to the rapid deterioration of the health state of the affected patients, resulting in a high risk of vascular hyperpermeability and multiorgan failure, and death in many patients [5, 6].

A number of authors had presented experimental data that established the role of insulin in glucose homeostasis and anti-inflammatory control influence. For instance, pretreatment with insulin had been shown to constrain the expression of pro-inflammatory cytokines through the inhibition of NF-κB activation, thus alleviating in vitro and in vivo inflammatory response [7, 8]. In addition, hyperinsulinemia was found to enhance antiviral immunity through direct stimulation of CD8+effectors T cell function [9]. Insulin requirement is believed to parallel illness severity in critically unwell COVID-19 patients [10]. Therefore, patients admitted to hospitals for severe COVID-19 may need modifications to their diabetes therapy, including the withdrawal of ongoing treatments and the initiation of insulin therapy. Such a decision should be based on the severity of COVID-19, nutritional status, actual glycemic control, the risk of hypoglycemia, renal function, and drug interactions. Although insulin treatment has been recommended for patients having diabetes with severe COVID-19, it remains to be determined whether the significance of the insulin treatment outcome is solely due to the control of blood sugar level, or mainly owed to the improvement of inflammatory condition reflected in the noticed inhibition of inflammatory mediators.

The effect of IL-6 on the disease course is presently indistinct; whether its elevation is detrimental or beneficial for COVID-19 is controversial [11]. When tested experimentally, IL-6 can either suppress or facilitate viral replication [12]. Hence, it is now more imperative than ever to explore this further by estimating IL-6 and exploring its association with other inflammatory modulators such as insulin.

The present study was undertaken to explore whether insulin has a modulatory role that leads to changes in the COVID-19 inflammatory state. To evaluate this, the study assessed the association of insulin with IL-6, taking into consideration that it is a potential treatment target for the cytokine storm, the common feature in COVID-19 severe patients.

## Materials and methods

This is a manuscript that represents a prospective study conducted on 60 patients admitted to the ICU of EL-Hawari General Hospital and El Marj Hospital in the Eastern part of Libya. These two units of the two hospitals were assigned to care for severe COVID-19 patients, hence all cases recruited for the investigation were admitted to these hospitals during the period extending from January 18, 2021, till March 22, 2021. Patients included in the study were those who were alive and still hospitalized on the day of the second sample collection, i.e., after seven days of hospitalization. In addition, none of the patients received antiviral therapy, and the administered drugs were aimed at mitigating the symptoms and improving the vital signs. Before commencing the study, we acquired ethical approval from the Research Ethics Board of Benghazi Medical Center. The approval letter carried the number 1.44.17, and it was issued on January 12th, 2021.

All cases were clinically diagnosed with the aid of chest X-ray (CXR), and certain cases were confirmed by immunoassay (IgG-IgM) or reverse transcription-polymerase chain reaction (RT-PCR). Except for insulin and IL-6, the clinical and laboratory data of the patients included in the study were collected from the file of the patient. Blood samples for estimating insulin and IL-6 were collected on the first day and after seven days of hospitalization.

All patients were asked for participation consent before they were included in the study. Patients’ data were recorded for use in this study using a data sheet (see Appendix) that we constructed for this purpose.

Patients were considered diabetic, according to the definition established by the World Health Organization diagnostic criteria: fasting plasma glucose ≥126 mg/dL or 2 h plasma glucose ≥200 mg/dL.

Aliquots of patients' blood collected by the hospital staff for routine laboratory tests were used by our team for IL-6 and insulin estimation. Insulin and IL-6 were estimated using the kits, Elecsys IL-6 and Elecsys Insulin (Roche Diagnostics, Germany).

In view of the fact that almost all our patients had high levels of IL-6, to answer whether IL-6 level had an effect on the proportion of patients who clinically improved and were eventually discharged compared to those who expired, the values of IL 6 estimates taken on admission day were subtracted from the values taken on the seventh day. Those that produced positive differences were referred to as low IL-6, and those that produced negative differences were referred to as high IL-6.

The quantitative data of the variables estimated for this study were expressed as means and standard deviations. In order to test the data for normal distribution, the Kolmogorov-Smirnov and Shapiro-Walk test were used. The variables were proven to be out of normal distribution. Hence, for comparing each variable at the two time points (on day one and after one week of hospitalization), the appropriate non-parametric test of Wilcoxon was selected assuming 95% confidence for continuous variable and Pearson’s chi-square test for categorizing variables association.

For statistical analysis, the SPSS version 28 (International Business Machines Corporation, Armonk, NY, USA) was used.

## Results

**Figure 1:**
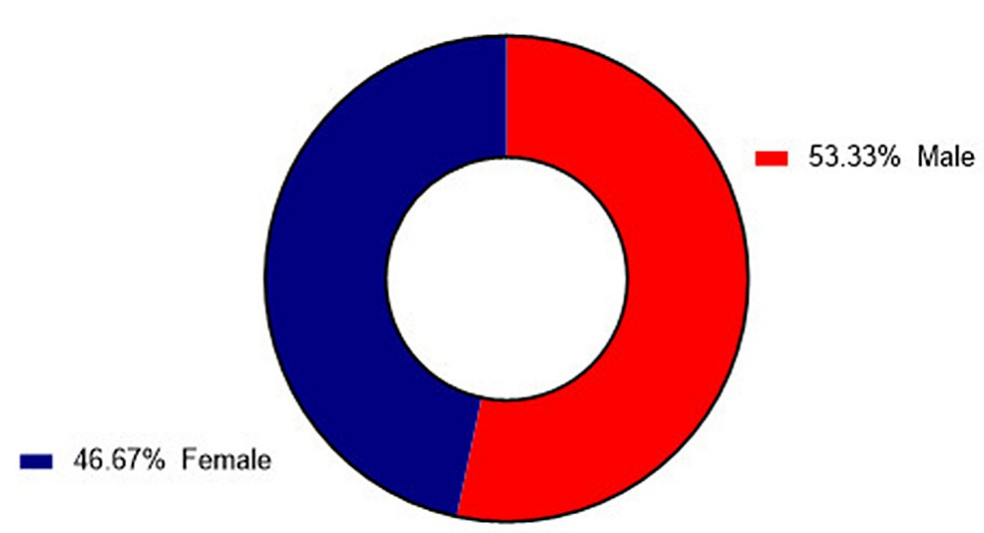
Male and female gender distribution of ICU admitted COVID-19 patients

A total of 60 severe COVID-19 patients were included in this study. Their mean age was 66.12 ± 12.79 years (range: 37-90); 32 patients (53.3%) were males and 28 (46.7%) were females (Figure 1).

The patients were admitted following the clinical diagnosis evidence based on the case history and the symptoms, which are commonly observed in COVID-19 patients. The confirmation of the diagnosis was established by chest X-ray for all the patients, immunoassay (IgG/IgM) for 18 patients (30%), and RT-PCR for 15 patients (25%) (Table 1).

**Table 1:** Mode of COVID-19 diagnostic confirmation CXR: Chest X-ray; RT-PCR: Reverse transcription-polymerase chain reaction.

Technique	Number of patients	Percent%
Clinically with CXR	60	100
Immunologically (IgG, IgM)	18	30
RT-PCR	15	25

The history of patients' comorbidity is depicted in Table 2. The most common chronic diseases coinciding with COVID-19 were diabetes mellitus (38 patients [63.3%]) and hypertension (34 patients [56.7%]). However, the less common comorbid diseases documented in our study included cardiovascular disease (10 patients [16.7%]), lung disease (nine patients [15%]), cerebrovascular accident (five patients [8.3%]), thyroid disease (four patients [6.7%]), kidney disease (three patients [5%]) and malignant neoplasm (two patients [3.3%]). Moreover, nine of the patients (15%) were newly diagnosed diabetics discovered upon admission, and the unfortunate in-hospital death was as high as 43 cases (71.7%).

**Table 2:** Chronic diseases comorbid with COVID-19 in the study patients

Comorbid disease	Number of patients	Percent %
Diabetes mellitus	38	63.3
Hypertension	34	56.7
Cardiovascular disease	10	16.7
Lung disease	9	15
Cerebrovascular accident	5	8.3
Thyroid disease	4	6.7
Kidney disease	3	5
Malignant neoplasm	2	3.3
Patients death	43	71.7

**Figure 2:**
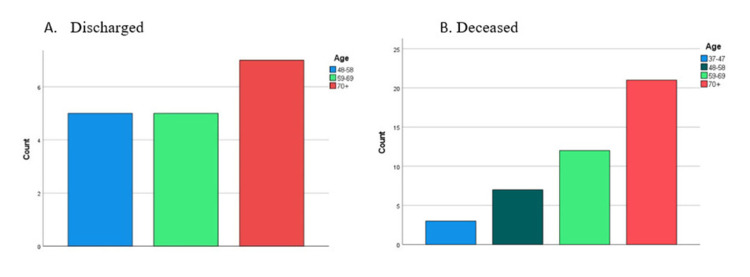
The discharged versus deceased patients count among the different age categories of COVID-19 admitted subjects

**Figure 3:**
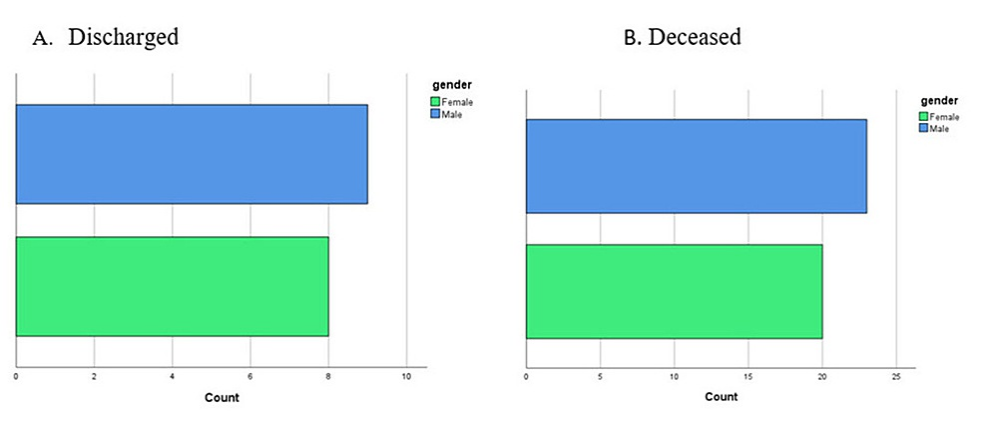
Gender differences in the number of discharged versus deceased COVID-19 patients

With reference to the outcome of the illness, older patients constituted higher proportions among those deceased during hospitalization and those discharged (Figure 2A, 2B). On the other hand, fewer COVID-19 ICU hospitalized females survived and were eventually discharged compared with males (Figure 3A), and similarly fewer females were deceased compared with males (Figure 3B).

**Figure 4:**
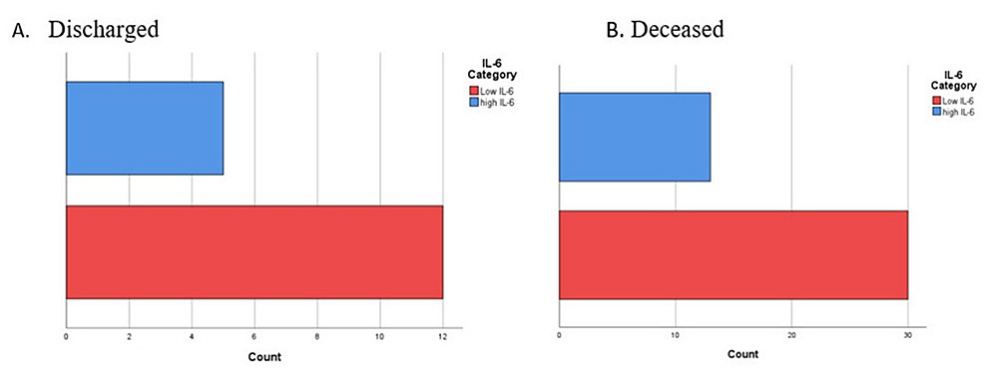
The proportion of discharged and deceased cases among the enlisted COVID-19 patients with high versus low IL-6 levels

The patients' IL-6 and insulin levels were both higher than the normal level at both time points, i.e., on the admission day and after seven days of hospitalization. Curiously enough, patients with lower IL-6 made a higher proportion among those deceased, but at the same time, they made a higher proportion among those discharged (Figure 4A, 4B). There was a noticeable increase in IL-6 levels comparing the second measurements mean (64.45 ± 42.68) to that taken upon admission (46.39 ± 68.58). On the contrary, insulin's estimates on the second time point were lower when comparing their mean to the mean of those taken on the admission day (Table 3).

**Table 3:** Wilcoxon signed-test comparing the means at the two time points of sample collection, the first estimates with the second of insulin and IL-6

Inflammatory Modulator	Mean	Median	SD	Z value	P-value
Insulin 1^st^ (mIU/L)	159.86	67.36	276.55	1.170	.09
Insulin 2^nd^ (mIU/L)	109.09	107.60	82.67		
IL-6 1^st^ (Pg/ml)	46.39	22.57	68.58	3.026	.001
IL-6 2^nd^ (Pg/ml)	64.45	72.46	42.68		

To investigate the hypothesis put forward in this study, we tested the association of insulin with IL-6 using chi-square (X^2^) test at 95% confidence. The test showed that there were significant associations between insulin and IL-6 (*P*=.002) using the estimates taken upon admission, and this association continued to be significant when tested using the estimates of the two variables after seven days of hospitalization (*P*=.010). The X^2^ results are shown in Table 4.

**Table 4:** Insulin and IL-6 association at the first and second time point of sample collection

	df	Pearson Chi-Square	Asymp. Sig. (2-sided)
Insulin 1^st^	1	9.600	.002
IL-6 1^st^			
Insulin 2^nd^	1	6.667	.010
IL-6 2^nd^			

## Discussion

The outcome of SARS-CoV-2 infection with its ever-increasing global mortality is believed to be related to the phenomenon known as the cytokine storm. This is thought to be the cause of the virus's severe acute respiratory syndrome and multi-organ injuries in critically ill patients. Patients with comorbidity such as type 2 diabetes (T2D) and hypertension are reported to develop the more severe form of the disease. However, although essential, it is unclear whether higher levels of blood insulin are beneficial for patients with COVID-19, and if such levels result in lower inflammatory markers.

In this prospective study, we show that among the critically ill patients hospitalized for COVID-19, a history of diabetes was the most common comorbid disease. With a prevalence of 63.3% among our COVID-19 patients, diabetes could be a major factor that increased the reported death rate. The high rate of diabetes seen in this study corresponds with the high prevalence of diabetes in the Libyan population. According to the WHO's report of 2016, the percentage of Libyan diabetics was 13.7% [13]. Furthermore, the trend in the incidence of type 2 diabetes is predicted to be on the rise as urbanization and life expectancy grow. Almost, 80% of Libyans live in urban areas [14]. This can be further complicated by the changes in lifestyle, such as the shift of food preference towards a more Westernized diet, and decreasing physical activity, which is associated with increased prevalence of conditions such as obesity [14].

During the surge of the COVID-19 pandemic, the intensive care unit of both EL Hawari General Hospital and El Marj Hospital were reserved for the care of severe cases and critically ill patients. This, in addition to the high percentage of diabetes, could explain the high mortality rate recorded among them (71.7%). Similarly, in Jin Yin-tan Hospital, in Wuhan, China, it was found that 32 (61.5%) patients died after 28 days of intensive care [15]. Moreover, in a large study in China that included 72,314 patients the percentage of deaths among hospitalized critically ill patients was 49.0% [16].

Although reasonably criticized [17], COVID-19 severity is believed to be influenced by the cytokine storm and viral evasion of cellular immune responses [18]. In addition, the persistent rise of inflammatory factors is believed to be associated with the increased risk of ARDS, disseminated intravascular coagulation (DIC), hypercoagulation, and multi-organ failure [19]. In our study, the evaluated IL-6 showed a significant increase at the time of admission compared to the normally documented levels and an upsurge after seven days of hospitalization, which could raise the plausibility that its changes during the course of the disease are related to the case advance, severity and possible outcome. Furthermore, this persistence of elevation in IL-6 was despite the treatment regimens including dexamethasone. Other authors who evaluated inflammation in COVID-19 patients presented higher levels of IL-6 in patients admitted to ICU and in those who did not survive [20, 21]. Similarly, other researchers had reported that hyperglycemic patients had greater levels of IL-6 during hospitalization for COVID-19 [22]. Furthermore, intensive insulin therapy has been shown to reduce mortality by 34%, and bloodstream infections by 46% during hospitalization [23], and those with hyperglycemia who received insulin infusion showed a lower risk of severe disease than those who did not receive insulin infusion [22].

To test the association of insulin with IL-6, and how this association progressed following hospitalization and treatment, we presented the levels of significance of this positive association (Table 4). Although insulin has been shown to exhibit anti-inflammatory action [17], it remains to be seen whether this is true for COVID-19 patients or contrary to that IL-6 increases insulin resistance [24], and hence its anti-inflammatory effects are overcome [25].

Attempting to control inflammation, dexamethasone given to patients with COVID-19 during the recovery trial reported by Horby et al. was found to affect mortality outcome [26]. Hence, immune modulation could be useful for the control of the cytokine storm. In the current study, if insulin is properly adjusted and its action boosted, its association with IL-6 may turn out to be a beneficial routine for critically ill and diabetic COVID-19 patients. Furthermore, in critically ill patients who had never had diabetes, hyperglycemia due to insulin resistance is common, and β cell damage caused by the virus would lead to insulin deficiency. In our study, 15% of the COVID-19 admitted patients were diagnosed with diabetes upon admission.

It is therefore imperative to determine whether insulin may be utilized as an anti-inflammatory drug, and whether insulin can control IL-6 and other inflammatory mediators. Although insulin's involvement as an anti-inflammatory factor has been described many years back [24], its efficacy for COVID-19 patients in terms of cytokine storm relief needs further investigation.

Immune dysregulation is reasonably believed to be driven by IL-6 level changes in patients with COVID-19, characterized by a cytokine storm. Scientific evidence shows that high serum IL-6 results in immune defects such as lymphopenia, impaired lymphocyte cytotoxicity, and endothelial activation. Some studies showed that treatment with IL-6 inhibitors such as tocilizumab can partially control such faulty immune states [27, 28]. Other studies demonstrated that elevated IL-6 concomitantly with raised CRP were highly expected to indicate respiratory failure [29]. In the present study, all other inflammatory markers showed high levels at admission (Data not presented).

Several limitations were encountered during the study. Firstly, other inflammatory markers such as ESR, CRP and WBC were measured at times suitable for patients’ condition and as doctors on duties see fit their needs for proper patient follow-up. Therefore, we were unable to utilize such data for this report as there were differences in the time of collection.

## Conclusions

Libyan patients with severe COVID-19 admitted to the ICU showed high levels of inflammatory markers, including the main target of the study, IL-6. Despite seven days of treatment that included 6 mg of dexamethasone, the levels of IL-6 remained on the rise. Hence, the patients were at high risk for developing the complications of the severe form of the disease with its fatal outcome. While insulin is believed to have an anti-inflammatory effect, in the current study, the level of insulin was shown to be reduced after seven days of hospitalization and treatment. We believe that considering insulin on its own as a positive effector for inflammation inhibition requires a deeper look and larger studies, possibly in less severe cases, where the outcome of treatment can be controlled to a larger extent. Furthermore, assessment of the efficiency of insulin in such patients should be considered.

## Figures and Tables

**Figure 5:**
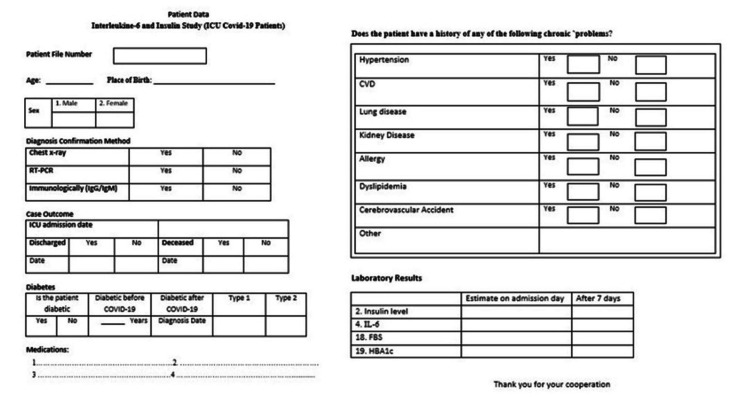
Data sheet used for collecting patients' history and lab results
